# Advances and Perspectives in Applying Deep Learning for Drug Design and Discovery

**DOI:** 10.3389/frobt.2019.00108

**Published:** 2019-11-05

**Authors:** Celio F. Lipinski, Vinicius G. Maltarollo, Patricia R. Oliveira, Alberico B. F. da Silva, Kathia Maria Honorio

**Affiliations:** ^1^Departamento de Química e Física Molecular, Instituto de Química de São Carlos, Universidade de São Paulo, São Carlos, Brazil; ^2^Faculdade de Farmácia, Universidade Federal de Minas Gerais, Belo Horizonte, Brazil; ^3^Escola de Artes, Ciências e Humanidades, Universidade de São Paulo, São Paulo, Brazil; ^4^Centro de Ciências Naturais e Humanas, Universidade Federal do ABC, Santo André, Brazil

**Keywords:** artificial intelligence, deep learning, medicinal chemistry, drug design, drug discovery

## Abstract

Discovering (or planning) a new drug candidate involves many parameters, which makes this process slow, costly, and leading to failures at the end in some cases. In the last decades, we have witnessed a revolution in the computational area (hardware, software, large-scale computing, etc.), as well as an explosion in data generation (big data), which raises the need for more sophisticated algorithms to analyze this myriad of data. In this scenario, we can highlight the potentialities of artificial intelligence (AI) or computational intelligence (CI) as a powerful tool to analyze medicinal chemistry data. According to IEEE, computational intelligence involves the theory, the design, the application, and the development of biologically and linguistically motivated computational paradigms. In addition, CI encompasses three main methodologies: neural networks (NN), fuzzy systems, and evolutionary computation. In particular, artificial neural networks have been successfully applied in medicinal chemistry studies. A branch of the NN area that has attracted a lot of attention refers to deep learning (DL) due to its generalization power and ability to extract features from data. Therefore, in this mini-review we will briefly outline the present scope, advances, and challenges related to the use of DL in drug design and discovery, describing successful studies involving quantitative structure-activity relationships (QSAR) and virtual screening (VS) of databases containing thousands of compounds.

## Artificial Intelligence and Machine Learning in Drug Discovery and Design

In the last decades, we have experienced a revolution in data science in terms of the huge amount of data to be analyzed (era of big data) and the availability of high-performance processors, in particular graphics processing unit (GPU) computing. In drug discovery, this scenario is not different: the large volume of data (chemical, biological, etc.) along with the automation of techniques have generated a fertile ground for the use of artificial (or computational) intelligence.

In medicinal chemistry, molecular features, such as physicochemical properties and other molecular descriptors can be related to the bioactivity level as molecular target affinity and to other pharmacokinetics properties (absorption, distribution, metabolism, and excretion) by using several computational methods. The results obtained by these techniques can assist the discovery and design of new drug candidates, if a suitable technique is chosen (Duch et al., 2007; Maltarollo et al., 2015, 2017).

The autonomous knowledge acquisition from the molecular properties of compounds requires the use of machine learning (ML) techniques, such as *k-*nearest neighbors (kNN), partial least squares (PLS), and artificial neural networks (ANN) (Gertrudes et al., 2012; Lavecchia, 2015; Lima et al., 2016). ML is a branch of artificial intelligence (AI), focusing on giving computers the capability of learning from data, which is of great importance in drug discovery protocols nowadays. From IEEE, computational intelligence (CI) involves the theory, the design, the application, and the development of biologically and linguistically motivated computational paradigms. In addition, CI encompasses three main methodologies: neural networks (NN), fuzzy systems, and evolutionary computation. In particular, artificial neural networks have been successfully applied in medicinal chemistry. Among the methodologies comprised by CI, deep learning (DL) has attracted a lot of attention in several areas due to its generalization power and ability to extract features from data (Gawehn et al., 2016; Sharma and Sharma, 2018).

## Deep Learning

Deep learning methods can be described as a class of representation-learning techniques that are able to discover, from the raw data, multiple-level of representations of increasing complexity by composing non-linear models. In this structure, each module in a level transform its input into a higher, more abstract representation. In this context, the term “deep” is associated to the number of layers in the network—the more layers, the deeper the network (Goodfellow et al., 2016). Two popular deep architectures are Deep Belief Networks (DBNs) (Hinton et al., 2006) and Convolutional Neural Networks (CNN) (LeCun et al., 1990). Multi-task learning for deep architectures has also been of great interest in many domain applications due to its ability to generalize predictive models to new scenarios (Zhang et al., 2018). Such approaches will be briefly described as follows.

## Restricted Boltzmann Machines (RBMs) and Deep Belief Networks (DBNs)

A restricted Boltzmann machine (Smolensky, 1986) is a two-layer, undirected, bipartite graphical model where the first layer (or visible layer) consists of the input data, and the second layer (or hidden layer) consists of latent variables. In this architecture, there are no intra-layer connections within either the visible or the hidden layer and the connections between the layer nodes are bi-directional, so given an input vector we can obtain its latent feature representation and vice versa. In this sense, RBM is considered a generative model, which learns the probability distribution of input data and generates new data points.

**Figure 1:**
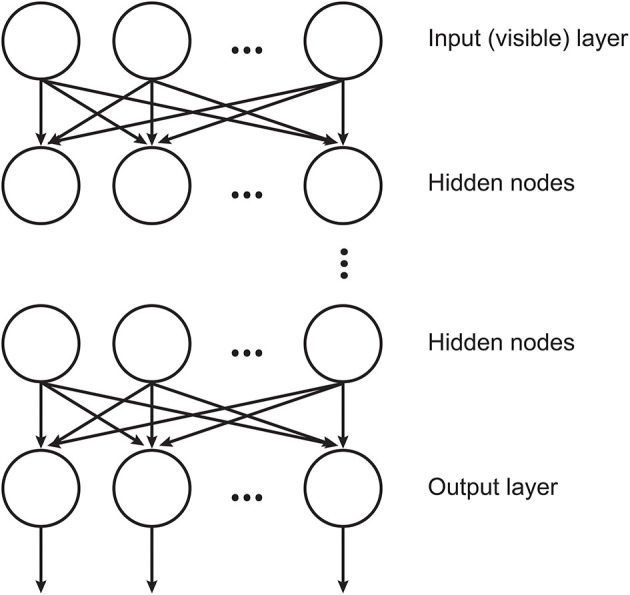
Illustration of the DBN structure, where the hidden layers are RBMs (adapted from Chen et al., 2012).

DBNs (Hinton et al., 2006) are neural networks consisted by RBMs stacked where each layer encodes statistical dependencies among the units in the layer below it. In such architecture, an RBM uses the previous layer's activations as inputs. The training procedure aims at maximizing the likelihood of the training data and involves two consecutive processes: the training of the individual layers, which is done in an unsupervised manner and the final fine-tuning process that is accomplished by a linear classifier. An illustration of DBN structure is displayed in Figure 1.

## Convolutional Neural Networks (CNNs)

**Figure 2:**
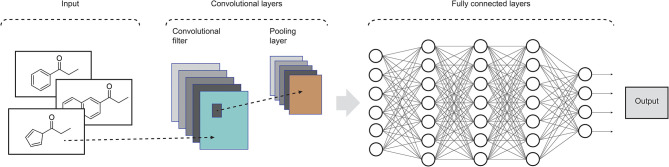
Illustration of the structure of a standard CNN. In drug design, the input data could be molecular structures or atom distances from molecular graphs (adapted from Rawat and Wang, 2017).

Widely applied in computing vision tasks, CNNs (LeCun et al., 1990) contain many layers that transform their input by applying convolution filters. In contrast to other deep learning structures, CNNs extract features of small portions of input images, which are called receptive fields. At each layer of this model, the input data is convolved with a set of filters, each generating a new feature map of data. Next, these features are subjected to a non-linear transformation and the same process is repeated for the remain convolutional layers. In order to increase the receptive field of subsequent convolutional layers and to make it invariant to small local deformations in the input, pooling layers are also incorporated in CNNs in order to aggregate pixel values of neighborhoods by using typically the max or mean operation. Fully connected layers are usually added at the end of the convolutional pipeline, feeding the activations in the final layer through a softmax function. CNNs commonly consist of several convolutional filters and many convolutional and pooling layers to enable the network to integrate the information extracted by different filters and various levels of abstraction (LeCun et al., 2015). Recent advances on CNN models can be found in Rawat and Wang (2017). An example of such architecture is shown in Figure 2.

## Multi-Task Learning

Multi-task learning (Caruana, 1997) aims at improving the generalization performance of multiple task predictors by jointly training them and thereby enabling learning multiple related objectives from a shared representation. As an example of task, we can consider the classification of chemical compounds as active or inactive according certain type of biological activity. DL naturally implements multi-task learning by allowing different related tasks to share the abstract features extracted during the learning process. Typically, multi-task learning can be helpful when the training data for a given task is small or imbalanced, which usually occurs in drug discovery applications. In a multi-task model, a task for which there is not sufficient information can acquire features from related tasks, then improving its performance. In a common setting, the training process for multi-task DL benefits from sharing the deep layers with all considered tasks, configuring the last layer to be task specific (Collobert and Weston, 2008). Some studies illustrating DL applications in medicinal chemistry will be described.

## DL in Medicinal Chemistry

In the drug discovery, DL has proved to be effective in handling large chemical libraries to provide predictive computational models, as well as modeling various properties of drug candidates, showing to be a highly relevant tool in virtual screening and quantitative structure-activity relationships (Pereira et al., 2016; Zhang et al., 2016). In protein engineering, DL can be applied to explore structures and functions of proteins, where it simulates interactions between them or with other molecules. DL has also been able to predict biological functions from fields of electron density and electrostatic potential, obtained directly from the raw data of these structures (Golkov et al., 2017). In analyses of gene expression data, using advanced sequencing technologies, DL has extracted knowledge from large amounts of genomic data to apply it in the genomic modeling for drug repurposing and in the precision medicine (Liang et al., 2015; Aliper et al., 2016).

DL has also been used to predict several different endpoints related to medicinal chemistry. From literature, we have found various studies on models to predict protein-ligand interactions, to score docking poses and to perform virtual screenings (Wallach et al., 2015; Pereira et al., 2016; Tian et al., 2016; Wen et al., 2017; Rifaioglu et al., 2018). Pharmacokinetic and toxicity parameters, such as aqueous solubility (Lusci et al., 2013) and specific toxicities (Unterthiner et al., 2015; Xu et al., 2015; Capuzzi et al., 2016) were also found as target-properties in prediction studies using DL models. So, applications of DL can be classified in ligand- and structure-based studies, as well as ligand-target interaction predictions. Some examples are described below.

## Ligand-Based Studies

Ligand-based approaches involve methodologies that used only information from the samples; in this case, chemicals with biological activity or other physicochemical property of interest (to be predicted). In general, molecular descriptors are used as input to construct models that relate chemical structure and biological activity. However, when DL is used for this purpose, graph representations of molecules (as graph convolution neural networks) are broadly employed.

An example of ligand-based study is performed by Lusci et al. (2013), which used a recursive deep learning architecture to predict aqueous solubility for four different benchmark datasets. This approach uses contracted graphs representation of molecules (called undirected graph recursive neural networks, UGRNN) that automatically extract and select structural features from molecules as learning step, differing from other methods in which the feature selection is often performed by human knowledge. In addition, the models generated by Lusci et al. outperformed other state-of-art works of aqueous solubility prediction for organic compounds.

Unterthiner et al. (2015) generated a deep neural network to predict several different binary toxic effects related to nuclear receptor and stress response pathways from Tox21 Data Challenge (National Center for Advancing Translational Sciences, 2015), which contains about 12,000 reported compounds with experimental data. The models were built by using the similarity between dataset compounds and known toxicophores, as well as molecular descriptors and Extended Connectivity FingerPrint (ECFP4) as input features. Several models were generated by combining parameters as number of hidden units and layers and the learning rates of backpropagation algorithm. Multi-task models outperformed single task models for almost all of 12 modeled endpoints. The results indicated that the deep learning-based model outperformed other generated models, winning the Tox21 Grand Challenge (Unterthiner et al., 2015; Mayr et al., 2016).

Xu et al. (2015) used three different datasets to predict drug-induced liver injury with UGRNN from the study performed by Lusci et al. (2013). The datasets were used to generate individual models as well combinations of databases. The model generated with a benchmark dataset predicted better than original model and the combined model outperformed the single models. Furthermore, the authors compared DL with a traditional neural network (NN) and the first approach outperformed NN.

Kearnes et al. (2016) applied molecular graph convolution deep neural networks to construct models for 259 datasets derived from the Tox21 challenge, “the maximum unbiased validation” dataset (MUV) PubChem BioAssay, and DUD-E. The deep neural networks were generated by including graph convolutional standard hyperparameters, atom features (as chirality, atomic charges, and aromaticity) and atom pair features (as bond type, and graph distance). The models were compared to logistic regression, random forests and a multitask neural network with two hidden layers trained with a circular fingerprint. In all cases, graph convolutional networks outperformed the logistic regression and random forests.

Altae-Tran et al. (2017) compared four convolutional architecture models with random forest using 100 trees and circular fingerprints as input to predict several datasets (Tox21, SIDER, and MUV). For both Tox21 and SIDER, the use of convolutional networks gives better results than random forest. For the MUV database, random forest performed better predictions, indicating that graph convolutional methods may have problems in the generalization of prediction in the case of new scaffolds. The authors also tried to perform transfer learning from models trained on the Tox21 datasets to predict observations from real patients (SIDER), but the predictability of the obtained models were not acceptable (AUC of a ROC curve about 0.5).

Ohue et al. (2019) proposed variations of graph convolutional networks by correcting the distances of atoms in a ring system and changing the treatment of atom pairs with the aim to represent better differences related to large interatomic distances of several conformations. The datasets employed in this study were Tox21, MUV, and PubChem BioAssay. The correction of atom distances on ring systems improved the accuracy of the models in comparison with a standard graph convolutional network (for all datasets). The correction of atom pair with large interaction distances also improved the accuracy of the models. These variations suggest that modifications in traditional graph convolutional deep neural networks, aiming to represent 3D effects from 2D structures, could be useful in the prediction of biological activities.

## Structure-Based Studies

Different from the previous approaches, structure-based techniques consider information from the samples and its molecular targets (for example, receptors, enzymes, and other structural or functional proteins). In this case, structure-based approaches can use interaction fingerprints (atom pairs from ligand-target complexes) and/or machine learning-based scoring functions to rank poses and hits (or classify the samples as active/inactive).

An example of this approach was performed by Wallach et al. (2015), in which the authors constructed deep learning models using DUD-E, ChEMBL-20 and an *in-house* version of ChEMBL-20 that contains experimentally inactive compounds instead decoys. The models were generated by using descriptors obtained from ligand-target complexes, such as the presence of certain atom types and functional groups into grid points of a 20 Å cubic box centered at center of mass of target binding site. These models were generated with a convolutional 3D layer method, which outperformed Smina scoring function (AutoDock Vina optimized version for scoring) in enrichment validations.

Pereira et al. (2016) proposed an approach (DeepVS) that uses docking results as input to train a deep neural network with the aim to distinguish active compounds from decoys by using features as atom types, atomic partial charges, amino acid types, and the distances from neighbors. This approach was employed and compared with DOCK 6.6 and AutoDock Vina 1.1.2 (ADV) using 40 molecular targets selected from DUD. DeepVS was also used to rank active molecules and decoys from eight randomly selected molecular targets from DUD-E. The results produced better AUC (area under the curve) values, which were calculated from enrichment plots indicating that this approach can be applied in virtual screenings with better performance than docking programs itself.

## Ligand-target Interaction Prediction

Finally, in this last category of studies the main objective is to predict the binding affinity of samples (ligands) with a specific molecular target. For example, Tian et al. (2016) generated models to predict compound-protein interactions by using DL. For this, compound-protein complexes were retrieved from STITCH database and considered as positive control; compounds and proteins were randomly generated as negative controls. This work considered, as input data, PubChem fingerprints that were taken as molecular descriptors, and protein features extracted from the Pfam database. After several model trainings, accomplished by varying the DL architecture, the best model performance was achieved by a network with four hidden layers and 2,000 unities in each layer. The results were compared to those obtained from logistic regression, support vector machines, and random forest models previously reported by other authors, indicating that DL outperformed all of them.

Wen et al. (2017) generated DBN models to predict drug-target interactions. The authors used drugs and targets retrieved from DrugBank as dataset and, as input features, Extended Connectivity Fingerprints (ECFP) and Protein Sequence Composition Descriptors (PSC) were employed to describe compounds and targets, respectively. The authors also compared the generated models with results obtained from random forest, decision tree and Naïve Bayesian approaches. The generated model outperformed the other techniques and, due to its high prediction performance in DrugBank dataset, the authors proposed that their tool could be employed in drug repurposing studies.

Rifaioglu et al. (2018) used ChEMBL database (only binding assays of molecular targets were kept in the dataset curation) as dataset to generate models. As features, 2D images of compounds were used as input for convolutional neural networks with the aim to classify images. Models were generated for enzymes, GPCRs (G protein-coupled receptors), ion channels, nuclear receptors, and other molecular targets. For all cases, DL outperformed logistic regression, random forest and support vector machines in classification of compounds as active or inactive.

Tsubaki et al. (2018) proposed a method that combines protein sequences and molecular fingerprints of ligands (from convolutional neural network and graph neural network, respectively) as vector input to predict protein-ligand interactions. The proposed method outperformed some techniques, such as kNN, random forest, logistic regression, and SVM. Finally, the authors concluded that the vectors obtained from the models correctly predicted important amino acid residues at the binding site responsible for drug-target interactions.

As proposed by Lee et al. (2019), the DeepConv-DTI method also uses the combination of protein sequences and molecular fingerprints of ligands to generate a fully connected layer, which represents the ligand-target complex in the predictive model. To generate the models, the authors used over than 32,000 drug-target complexes from DrugBank, KEGG, and IUPHAR databases. In addition, to evaluate the ability to predict binding affinity of samples into the molecular targets, PubChem BioAssay, ChEMBL, and KinaseSARfari were employed as test sets. The results indicated that deep neural networks outperformed similarity-based models and conventional representation of proteins.

## Conclusions

Due to the fast development of computing power and the generation of enormous amount of chemical and biological data, projects involving drug discovery have been benefited from artificial intelligence. Particularly, in the last decades, we have observed a significant increase in the number of studies using deep learning. Some applications of DL involve studies of quantitative structure-activity relationships (QSAR), virtual screening, drug repositioning and *in silico* prediction of pharmacokinectic properties (absorption, distribution, metabolism, and excretion–ADME) and toxicity. It is important to highlight that traditional techniques have been outperformed by DL in some applications related to drug design due some intrinsic characteristics of biological and chemical data, such as complexity, uncertainty, diversity, and high dimensionality. The main advantages of DL refer to the scale and the complexity of the neural networks used to build robust and predictive models, as well the flexibility in their architecture, allowing for adaptations to specific problems. Some drawbacks of applying DL include the limited number of data in some areas of study and the difficult interpretation of the chemical and biological mechanisms involved in the DL models. In summary, from the many applications of DL in drug design we can conclude that many advances have been observed in this area and new applications and methodologies have been developed every day, making this technique a reliable tool in the arsenal available for the discovery of new drug candidates.

## Author Contributions

All authors listed have made a substantial, direct and intellectual contribution to the work, and approved it for publication.

### Conflict of Interest

The authors declare that the research was conducted in the absence of any commercial or financial relationships that could be construed as a potential conflict of interest.
